# Integrated analysis of intestinal microbiota and metabolomic reveals that decapod iridescent virus 1 (DIV1) infection induces secondary bacterial infection and metabolic reprogramming in *Marsupenaeus japonicus*

**DOI:** 10.3389/fimmu.2022.982717

**Published:** 2022-09-16

**Authors:** Zihao He, Yunqi Zhong, Minze Liao, Linxin Dai, Yue Wang, Shuang Zhang, Chengbo Sun

**Affiliations:** ^1^ College of Fisheries, Guangdong Ocean University, Zhanjiang, China; ^2^ Aquatic Animals Precision Nutrition and High Efficiency Feed Engineering Research Center of Guangdong Province, Zhanjiang, China; ^3^ Guangdong Provincial Laboratory of Southern Marine Science and Engineering, Zhanjiang, China; ^4^ Guangdong Provincial Key Laboratory of Pathogenic Biology and Epidemiology for Aquatic Economic Animals, Zhanjiang, China

**Keywords:** decapod iridescent virus 1, *Marsupenaeus japonicus*, intestinal microbiota, metabolomic, secondary bacterial infection, metabolic reprogramming

## Abstract

In recent years, with global warming and increasing marine pollution, some novel marine viruses have become widespread in the aquaculture industry, causing huge losses to the aquaculture industry. Decapod iridescent virus 1 (DIV1) is one of the newly discovered marine viruses that has been reported to be detected in a variety of farmed crustacean and wild populations. Several previous studies have found that DIV1 can induce Warburg effect-related gene expression. In this study, the effects of DIV1 infection on intestinal health of shrimp were further explored from the aspects of histological, enzymatic activities, microorganisms and metabolites using *Marsupenaeus japonicus* as the object of study. The results showed that obvious injury in the intestinal mucosa was observed after DIV1 infection, the oxidative and antioxidant capacity of the shrimp intestine was unbalanced, the activity of lysozyme was decreased, and the activities of digestive enzymes were disordered, and secondary bacterial infection was caused. Furthermore, the increased abundance of harmful bacteria, such as *Photobacterium* and *Vibrio*, may synergized with DIV1 to promote the Warburg effect and induce metabolic reprogramming, thereby providing material and energy for DIV1 replication. This study is the first to report the changes of intestinal microbiota and metabolites of *M. japonicus* under DIV1 infection, demonstrating that DIV1 can induce secondary bacterial infection and metabolic reprogramming. Several bacteria and metabolites highly associated with DIV1 infection were screened, which may be leveraged for diagnosis of pathogenic infections or incorporated as exogenous metabolites to enhance immune response.

## Highlights

- DIV1 infection disrupts the shrimp intestinal mechanical barrier.
- DIV1 infection resulted in imbalance of oxidative and antioxidant capacity, decreased immune enzyme activity, and disordered digestive enzyme activity in shrimp.
- DIV1 infection caused secondary bacterial infections, including Photobacterium and Vibrio.
- DIV1 can cooperate with harmful bacteria to induce metabolic reprogramming.

## 1 Introduction

Viruses are an important part of marine ecosystems. With the development of genetic technology, more and more new marine RNA and DNA viruses have been discovered (1, 2). Despite their tiny size, viruses play a large role in marine ecosystems and food webs. On the one hand, marine viruses can infect a variety of oceans organisms, lysing their cells and releasing carbon and other nutrients that impact the food web (3). On the other hand, marine viruses often contain host-derived metabolic genes (i.e., auxiliary metabolic genes; AMGs), which are hypothesized to increase viral replication and alter ecosystem-level productivity through reprogramming host metabolism (4). To date, several crustacean viruses have been found to induce metabolic reprogramming to promote survival and replication, including white spot syndrome virus (WSSV) (5–8), Taura syndrome virus (TSV) (9) and IHHNV (10). In recent years, with global warming and increasing marine pollution, some novel marine viruses have become widespread in the aquaculture industry, causing huge losses to the aquaculture industry (11). Decapod iridescent virus 1 (DIV1) is one of the newly discovered marine viruses. Since China initiated the DIV1 surveillance in 2017, DIV1 has been detected in shrimp culture ponds in several provinces (12). In addition to farmed shrimp, in 2020, Srisala et al. also detected DIV1 in wild populations of *Penaeus monodon* in the Indian Ocean (13). Up to now, DIV1 has been known as a highly lethal virus with global risk of transmission, capable of infecting freshwater and marine crustaceans, including *Marsupenaeus japonicus* (14).

Kuruma shrimp *M. japonicus* is widely distributed in the Indo-Western Pacific region and the East and South China seas (15). Due to its high economic value, strong environmental adaptability and suitability for long-distance transportation, it has now become one of the most farmed prawns in China (16, 17). Our previous study demonstrated that *M. japonicus* showed obvious clinical symptoms after DIV1 challenge, including empty stomach and intestine, atrophy of the hepatopancreas with yellowing, red body and soft shell. The shrimp mortality rate increased as the virus dose increased, and reached 100% mortality after at 76 hpi injected with 3.95 × 10^9^ copies/μg DNA DIV1 inoculum (14). Through mRNA-seq and miRNA-seq analyses and their association analysis, we preliminarily revealed the hemocyte and intestinal immune response of *M. japonicus* to DIV1 infection (11, 14), and proposed that DIV1 can promote the Warburg effect by regulating host miRNA and mRNA expression. The Warburg effect was also observed in DIV1-infected *Litopenaeus vannamei* and *Penaeus monodon*. However, only the miRNA and mRNA expression profiles under DIV1 infection were revealed, the deep mechanism of the immune response needs further analysis, especially the intestinal immunity.

As an important immune and digestive organ of shrimp, the intestine and symbiotic microorganisms together constitute a complex ecosystem, which plays an important role in maintaining the function of the shrimp immune system. The intestinal immune system of shrimp is mainly composed of three parts, including the mechanical barrier formed by the tight junction and adhesion junction of intestinal mucosal cells, the innate immune barrier formed by intestinal hemocytes and their secreted immune factors, and the biological barrier formed by the intestinal microbiota and their secretions (18). The potential cooperation of bacteria and viruses in promoting disease development has received extensive attention from researchers in recent years. Numerous studies have shown that viral infection can alter the composition and function of the shrimp intestinal microbiota, induce secondary bacterial infection, and impair host immunity (19–21). However, similar to other invertebrates, shrimp lack specific immunity and cannot acquire antibodies through vaccination. Therefore, regulating host metabolic reprogramming by exogenous metabolites to enhance host immunity and ability to withstand environmental has become one of the best strategies for shrimp to resist viral infection (22–24). It will be interesting to explore the effect of metabolic reprogramming on host intestinal metabolome and microbiota and the consequence on immune response. However, there are no reports on the effects of DIV1 infection on the intestinal metabolome and microbiome of shrimp.

To gain a more comprehensive understanding of the interaction between DIV1 and shrimp, this study is the first to investigate the changes of intestinal microbiota and metabolites of *M. japonicus* under DIV1 infection through the integrated analysis of intestinal microbiome and metabolomics, demonstrating that DIV1 can induce secondary bacterial infection and metabolic reprogramming, and several highly related bacteria and metabolites highly associated with DIV1 infection were screened. The results are beneficial to provide a theoretical basis for virus control technology.

## 2 Materials and methods

### 2.1 Shrimp and rearing conditions

Healthy *M. japonicus* with an average body weight of 10.5 ± 1.6 g were randomly obtained from a local farming pond in East Island Marine Biological Research Base, Guangdong Ocean University (Zhanjiang, China). The shrimp were randomly sampled and tested by PCR to ensure that they were free from WSSV, IHHNV, and DIV1. The detection method was consistent with the previous study (25). Every 30 *M. japonicus* were acclimatized in 0.3-m^3^ tanks with aerated and filtered seawater (salinity 30‰, pH 7.5, temperature 28°C) for 7 days before the DIV1 challenge experiment. Commercial feed was provided to the shrimp three times per day at a feeding rate of 5% of their body weight, and nearly 90% of the seawater was exchanged per day.

### 2.2 DIV1 challenge and sample collection

After 7 days of acclimation, the healthy shrimp were divided into two groups: the PBS-injected group and the DIV1-infected group. Each group included three replicate tanks, and each tank containing 30 individuals. Base on LC_50_ test results from previous studies (14), we set the DIV1 injection concentration of this study as 3.95 × 10^9^ copies/μg DNA. Each *M. japonicus* from the DIV1-infected group was intramuscularly injected with 50 μL of DIV1 inoculum, while each *M. japonicus* from the PBS-injected group was intramuscularly injected with 50 μL of phosphate-buffered saline (PBS; pH 7.4). The methods of viral inoculum preparation and quantification can be found in previous studies (26).

Based on previous research (14), the *M. japonicus* at twenty-four hour post-injection (hpi) were collected as samples under an aseptic condition. The intestines and their contents from 3 random individuals in the same group were combined as one sample and immediately frozen in liquid nitrogen before storing at -80°C until experimental analysis. In detail, each group had 6 samples were used for enzyme activity analysis, 6 samples were used for intestine microbiome analysis and 3 samples were used for intestinal metabolomics analysis. For histological analysis, the intestines of 6 shrimp per group were used to assess intestine tissue damage. Intestines of these shrimp were fixed in 10% formalin.

### 2.3 Histological analysis

After fixed in 10% formalin at 4°C for 24 h, the fixed intestine were dehydrated in gradient ethanol, hyalinized in xylene, and embedded in paraffin wax. Next, the paraffin blocks were sectioned at 5-μm thickness. The sections were collected on glass slides and stained with hematoxylin and eosin (H&E). The intestinal sections were examined by a microscope (Olympus, Nikon, Tokyo, Japan). The electronic images were further analyzed using ImageJ software to assess the dimensions of intestinal villus height and muscle thickness.

### 2.4 Enzyme activity analysis

Enzymatic biomarkers of functional responses in the intestine were measured using commercial detection kits (Jianglai Bioengineering Institute, Shanghai, China) according to protocols of the manufacturer. Prior to analysis, 6 samples in each group were homogenized in pre-chilled PBS (1:9 dilution) and then centrifuged for 10 min (4°C and 5,000 × g) to obtain the supernatant for further use. Superoxide dismutase (SOD), catalase (CAT) and lysozyme (LYZ) activities were used to reflect the nonspecific immunity in the shrimp intestine. α-Amylase (α-AMS), lipase (LPS) and trypsin (TPS) activities were used to reflect the digestive function of shrimp.

### 2.5 Intestinal microbiome analysis

Total bacterial DNA was extracted by the *EasyPure* ^®^ Marine Animal Genomic DNA Kit (TransGen Biotech, China) following the manufacturer’s directions. The concentration and purity of total DNA were determined by SimpliNano (GE Healthcare, United States) and 1% agarose gels. The primers pair 515F (5′-GTGCCAGCMGCCGCGG-3′) and 806R (5-′GGACTACNNGGGTATCTAAT-3′) were used to amplify the V4 hypervariable region of 16S rRNA gene, which was modified with a barcode tag with a random 6-base oligos. The PCR amplification reaction was performed with a 25 μL reaction mixture containing 12.5 μL rTaq mix (Takara Biotechnology (Dalian) Co., Ltd.), 1 μL of each primer, 1μL template and 9.5 μL ultrapure water. The reaction cycle parameters were as follows: an initial denaturation step of 95°C for 3 min; 30 serial cycles of a denaturation step of 95°C for 45 s, annealing at 55°C for 45 s, and extension at 72°C for 45 s; and a final extension step of 72°C for 10 min.

PCR amplification reaction system and parameters can refer to previous studies (27). After the PCR products were purified and mixed in equidensity ratios, sequencing libraries were constructed, and then sequenced by BGI (Shenzhen, China) with the Illumina Genome Analyzer technology. All raw sequencing data of intestinal microbiota was submitted to the Sequence Read Archive (SRA) (accession: PRJNA720257).

Sequences from raw data were analyzed and filtered by QIIME (v1.8.0). Sequence analysis was performed with UPARSE software (v7.0.1090), and the operational taxonomic units (OTUs) were defined with ≥ 97% similarity. Chimeric sequences were identified with UCHIME (v4.2.40). Alpha diversity was calculated using mothur software with five metrics, including the observed OTUs, Chao1, ACE, Shannon and Simpson indexes. Rarefaction curves were generated based on these metrics. The shared and unique OTUs between two groups were figured out by a Venn diagram. Beta diversity index based on the phylogenetic relationship between OTUs was used to calculate the Unifrac distance (weighted and unweighted Unifrac) and the results of Beta diversity were showed through PCoA and UPGMA Phylogenetic Dendrogram. A bar plot of the microbial community was constructed at the phylum, family and genus level respectively. To study the functional characteristics of bacterial communities, Kyoto Encyclopedia of Genes and Genomes (KEGG) functions were predicted using the PICRUSt software.

### 2.6 Intestinal metabolomics analysis

Six intestinal samples replicates of shrimp from each group were used for metabolomic analysis. All the samples were taken from the refrigerator at -80°C and thawed in the refrigerator at 4°C, and metabolite extraction was performed using methanol and 2-chlorobenzalanine. Twenty microlitres of each sample was taken for quality control (QC), and the rest was used for LC-MS detection. Liquid chromatography was accomplished in a Thermo Ultimate 3000 system equipped with an ACQUITY UPLC^®^ BEH C18 (1.7 μm 2.1 × 100 mm, Waters, USA) column. Mass spectrometry was executed on a Q Exactive HF mass spectrometer (Thermo Fisher Scientific, USA). Data-dependent acquisition (DDA) MS/MS experiments were performed with HCD scans. Dynamic exclusion was implemented to remove some unnecessary information in the MS/MS spectra.

Peak-identification, peak-alignment and compound identification was conducted using Compound Discoverer software (v3.1). All the data were determined using quality control (QC) and quality assurance (QA). Partial least squares-discriminant analysis (PLS-DA) of the metabolomics data was performed using the R language ropls package. All the metabolites were classified according to Kyoto Encyclopedia of Genes and Genomes (KEGG) and Human Metabolome Database (HMDB). The PLS-DA model was used to determine the differential metabolites (DMs) between the PBS-injected group and the DIV1-infected group with the first principal component of variable importance in projection (VIP) values (VIP ≥ 1) combined with a *q*-value ≤ 0.05. The DMs were annotated with KEGG pathway analysis to further identified the change characteristics of the functional metabolites related to the immunity of the shrimp.

### 2.7 Correlation analysis of intestinal bacteria and DMs

Canonical correlation analysis and spearman correlation analysis were employed to reveal the correlation between significantly altered intestinal bacteria and intestinal DMs, and the results were shown with scatterplots and heat maps. The thresholds for correlation coefficients and *p*-values were not set. *p* < 0.05 was regarded as statistically significant, *p* < 0.01 was regarded as very significant, and *p* < 0.001 was regarded as extremely significant.

## 3 Result

### 3.1 Intestinal histological changes

**Figure 1:**
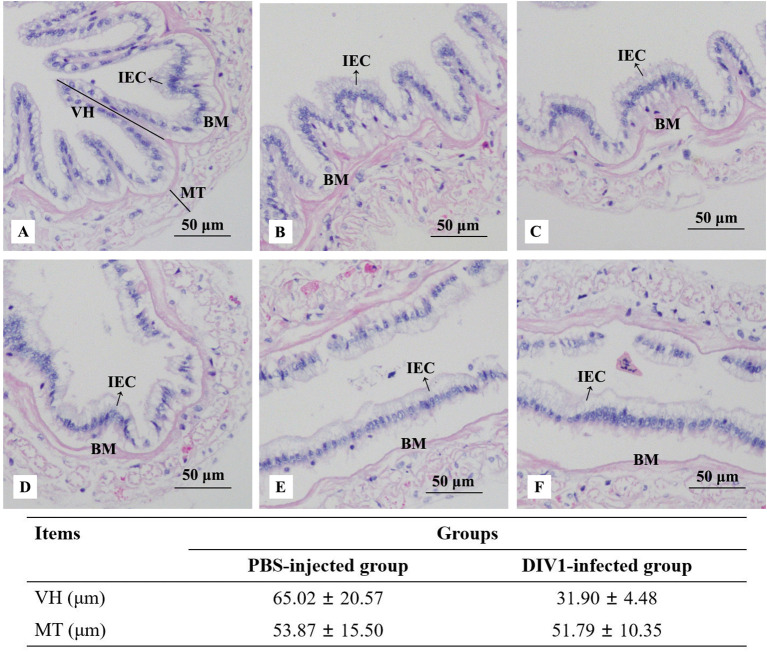
Photomicrographs of intestinal sections in the PBS-injected group **(A-C)** and the DIV1-infected group **(D-F)**. IEC, intestinal epithelial cells; BM, basement membrane; VH, villus height; MT, muscle thickness.

Histological analysis of the intestinal sections from the PBS-injected group and the DIV1-infected group are shown in **Figure 1**. The H&E staining showed that no histological changes were observed in the PBS-injected group (**Figures 1A**). Contrastively, the microstructure of the intestine in the DIV1-infected group had lesions, some intestinal epithelial cells were detached from the basement membrane and destroyed (**Figures 1D**). Intestinal villus height and muscle thickness were measured to quantify the degree of intestinal damage. The results showed that the intestinal villus height in the PBS-injected group (65.02 ± 20.57 μm) was significantly higher than the DIV1-infected group (31.90 ± 4.48 μm) (*p* < 0.001), but the intestinal muscle thickness did not show significantly difference between the PBS-injected group (53.87 ± 15.50 μm) and the DIV1-infected group (51.79 ± 10.35 μm) (*p* > 0.05).

### 3.2 Immune and digestive enzymes activity abnormalities

**Figure 2:**
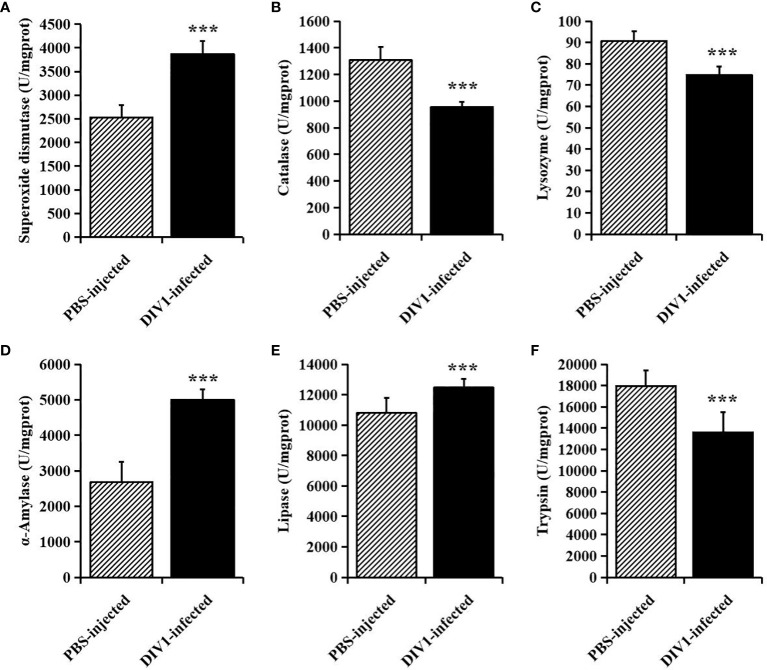
The activities of immune and digestive enzymes in the intestine of *M. japonicus* (mean ± SD). **(A)** Superoxide dismutase (SOD). **(B)** catalase (CAT). **(C)** lysozyme (LYZ). **(D)** α-Amylase. **(E)** lipase (LPS) and **(F)** trypsin (TPS). The statistically significant differences between the two groups were calculated by Student’s *t*-test (***p < 0.001).

At 24 hpi, the activities of SOD, CAT and LYZ in the intestine were detected to evaluate the effect of DIV1 infection on the nonspecific immunity of *M. japonicus* (**Figures 2A–C**), and the activities of α-AMS, LPS and TPS in the intestine were detected to evaluate the digestive function (**Figures 2D–F**). Compared with the PBS-injected group, the activity of SOD, α-AMS and LPS were significantly increased (*p* < 0.001), while the activities of CAT, LYZ and TPS were significantly decreased (*p* < 0.001). It was worth noting that, after infection with DIV1, both the immune and digestive enzymes in the intestine were extremely significantly altered (*p* < 0.001).

### 3.3 Intestinal microbiota changes

#### 3.3.1 Richness and diversity

**Figure 3:**
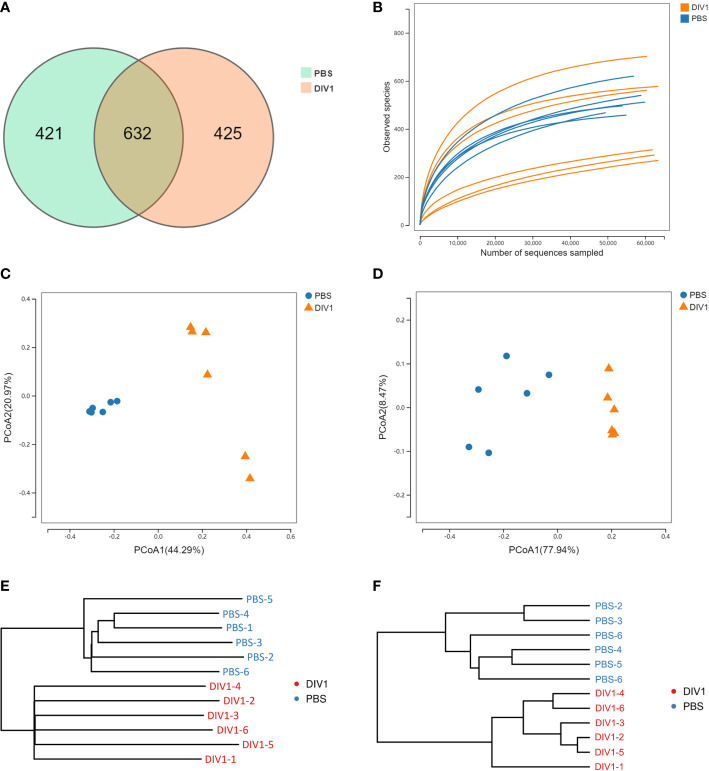
Intestinal microbial richness and diversity of *M. japonicus* after DIV1 infection. **(A)** Veen diagram showing the unique and shared OTUs of intestinal microbiota in the PBS-injected group and the DIV1-infected group. **(B)** Rarefaction curves of OTUs clustered at 97% sequence identity different samples. **(C)** PCoA plot with unweighted unifrac distance. **(D)** PCoA plot with weighted unifrac distance. **(E)** UPGMA Phylogenetic Dendrogram with unweighted unifrac distance. **(F)** UPGMA Phylogenetic Dendrogram with weighted unifrac distance.

A total of 711,264 high-quality sequences were generated from 12 intestinal samples. The clean reads ranged from 69,929 to 63,969, with an average of 59,272 clean reads per sample. After the alignment, the sequences of the PBS-injected group and the DIV1-infected group were clustered into 1,053 and 1,057 OTUs respectively with a 97% sequence similarity. Among them, there were 421 unique OTUs in the PBS-injected group, 425 unique OTUs in the DIV1-infected group, and 632 OTUs were the same in both the PBS-injected group and the DIV1-infected group (**Figure 3A**). A rarefaction curve analysis of the observed species per sample was sufficient (**Figure 3B**). To investigate the differences of species diversity and richness between two groups, the alpha diversity indexes were calculated, including observed OTUs, Chao1, ACE, Shannon and Simpson indexes, ranging from 270 to 715, 400.25 to 753.01, 424.50 to 765.22, 0.41 to 3.19 and 0.10 to 0.89, respectively (**Table 1**). Community richness indexes (observed OTUs, Chao1, ACE) were not significantly changed, while community diversity indexes (Shannon and Simpson) were significantly changed (*p* < 0.05). Beta diversity analysis was performed to comparative analyze the similarity and difference of intestinal microbial community in different groups. The PCoA with weighted and unweighted unifrac distance were further performed to confirm that intestinal bacteria in the PBS-injected group and the DIV1-infected group were clearly separated and samples from the same group were clustered closer (**Figures 3C, D**). UPGMA Phylogenetic Dendrogram with weighted and unweighted unifrac distance showed that all detected samples were divided into two main clades, and the intestinal microbial in the same group had a high degree of similarity (**Figures 3E, F**).

**Table 1:** Alpha diversity index analysis in the PBS-injected group and the DIV1-infected group.

Sample	Sobs	Chao1	ACE	Shannon	Simpson	Coverage
PBS-1	512	574.06	577.24	2.65	0.18	0.998304
PBS-2	497	529.76	538.83	2.06	0.37	0.998602
PBS-3	458	490.50	493.66	2.40	0.30	0.998809
PBS-4	541	640.29	666.17	2.60	0.21	0.997645
PBS-5	469	580.91	605.59	2.17	0.22	0.997116
PBS-6	621	678.58	703.00	3.18	0.10	0.997910
DIV1-1	703	736.11	747.55	2.73	0.27	0.998572
DIV1-2	292	470.22	589.70	0.37	0.90	0.997863
DIV1-3	314	450.56	537.92	0.74	0.80	0.998154
DIV1-4	562	643.01	639.31	2.10	0.34	0.998189
DIV1-5	270	404.72	430.81	0.76	0.64	0.998124
DIV1-6	578	611.13	614.32	2.04	0.43	0.998890
*p*-values	0.441	0.631	0.940	0.032	0.014	0.451

#### 3.3.2 Intestinal microbial composition

**Figure 4:**
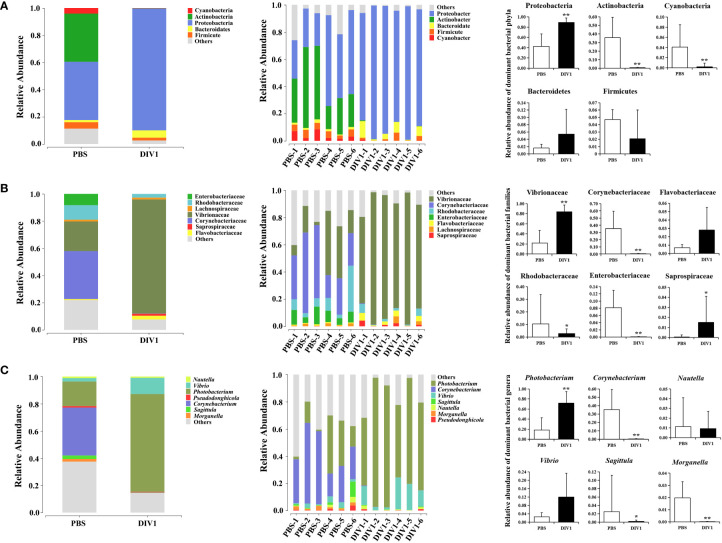
Structure and composition of the intestinal bacterial communities under DIV1 infection (mean ± SD). **(A)** Relative abundances of dominant bacterial phyla. **(B)** Relative abundances of dominant bacterial families. **(C)** Relative abundances of dominant bacterial genera.* Indicates a significant difference (*p < 0.05; **p < 0.01).

The taxa of dominant bacteria among the two groups were similar, while their abundance was altered significantly. At the phylum level, compared with the PBS-injected group, the relative abundance of Proteobacteria was significantly increased in the DIV1-infected group (*p* < 0.01), while the relative abundance of Actinobacteria and Cyanobacteria were significantly decreased (*p* < 0.01) (**Figure 4A**). At the family level, the relative abundance of Vibrionaceae and Sapropiraceae were significantly increased in the DIV1-infected group (*p* < 0.05), while the relative abundance of Corynebacteriaceae, Rhodobacteraceae and Enterobacteriaceae were significantly decreased (*p* < 0.05) (**Figure 4B**). At the genus level, differences were also observed. The relative abundance of *Photobacterium* was significantly decreased (*p* < 0.01), while the relative abundance of *Corynebacterium*, *Sagittula* and *Morganella* were significantly decreased (*p* < 0.05).

#### 3.3.3 Changes in the intestinal bacterial phylotypes

**Figure 5:**
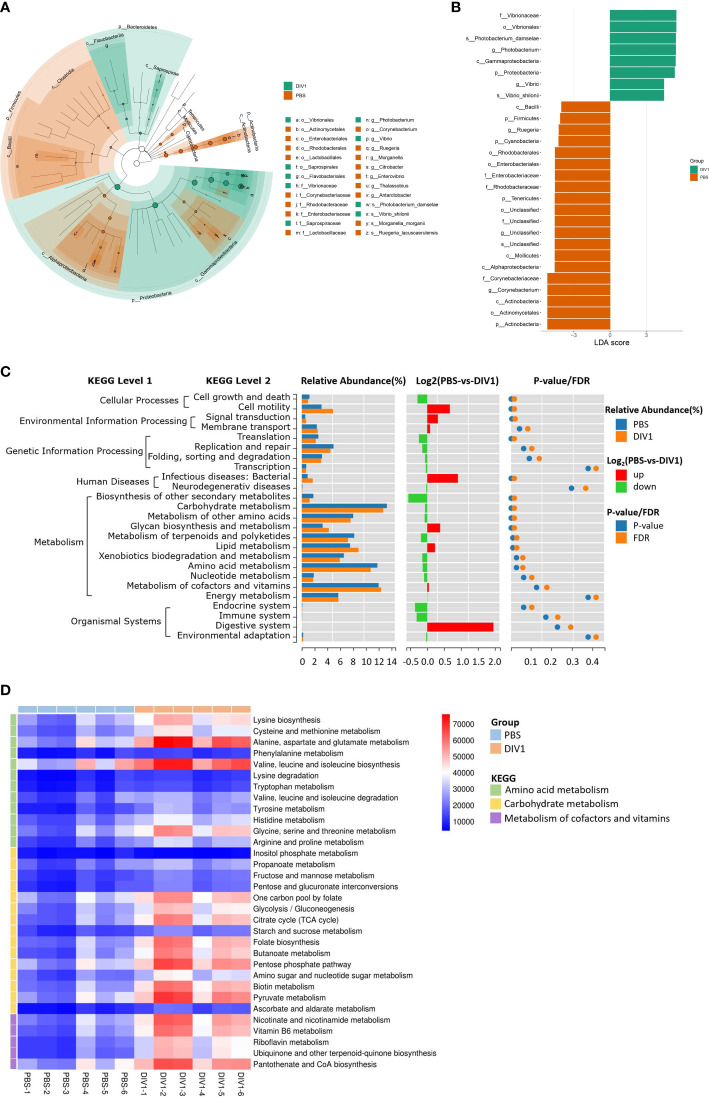
Intergroup variation, functional analysis of intestinal microbiota of *M. japonicus* after DIV1 infection. **(A)** LEfSe cladogram. **(B)** LDA score of LEfSe-PICRUSt. **(C)** Microbial metabolism prediction based on KEGG pathway analysis (KEGG level 2). **(D)** Abundance clustering heat map of predicted functions of intestinal microbiota in each sample (KEGG level 3).

LEfSe was employed to analyse the differential abundances of bacterial taxa in the two groups. In the cladogram, the families Vibrionaceae and Saprospiraceae were enriched in the DIV1-infected group, and Corynebacteriaceae, Rhodobacteraceae, Enterobacteriaceae and Lactobacillaceae were enriched in the PBS-injected group (**Figure 5A**). With an LDA score greater than 4.0, *Photobacterium* and *Vibrio* dominated in the DIV1-infected group, and Corynebacterium and Ruegeria dominated in the PBS-injected group (**Figure 5B**). The prediction function of the intestinal microbiota was analyzed using PICRUSt. Result showed that, in the KEGG level 2, the relative abundance of “carbohydrate metabolism”, “metabolism of cofactors and vitamins” and “amino acid metabolism” were the top 3 in the two groups (**Figure 5C**). It was worth noting that the relative abundance of “infectious diseases: bacterial” was significantly increased under DIV1 infection (*p* < 0.01). The distinct changes on a deeper resolution level within “Carbohydrate metabolism”, “Metabolism of cofactors and vitamins” and “Amino acid metabolism” were showed by the heatmap (**Figure 5D**). The results showed that the abundance value of KEGG level 3 term “alanine, aspartate and glutamate metabolism” and “valine, leucine and isoleucine biosynthesis” in the DIV1-infected group were significantly higher than the PBS-injected group (*p* < 0.01). The abundance value of some Warburg effect marker pathways and vitamin metabolism-related pathways were also significantly increased after DIV1 infection (*p* < 0.01), including “glycolysis/guconeogenesis”, “pyruvate metabolism”, “nicotinate and nicotinamide metabolism”, “vitamin B6 metabolism”, “riboflavin metabolism” and “pantothenate and CoA biosynthesis”.

### 3.4 Intestinal metabolic pattern alterations

#### 3.4.1 Multivariate analysis of the metabolite profiles

**Figure 6:**
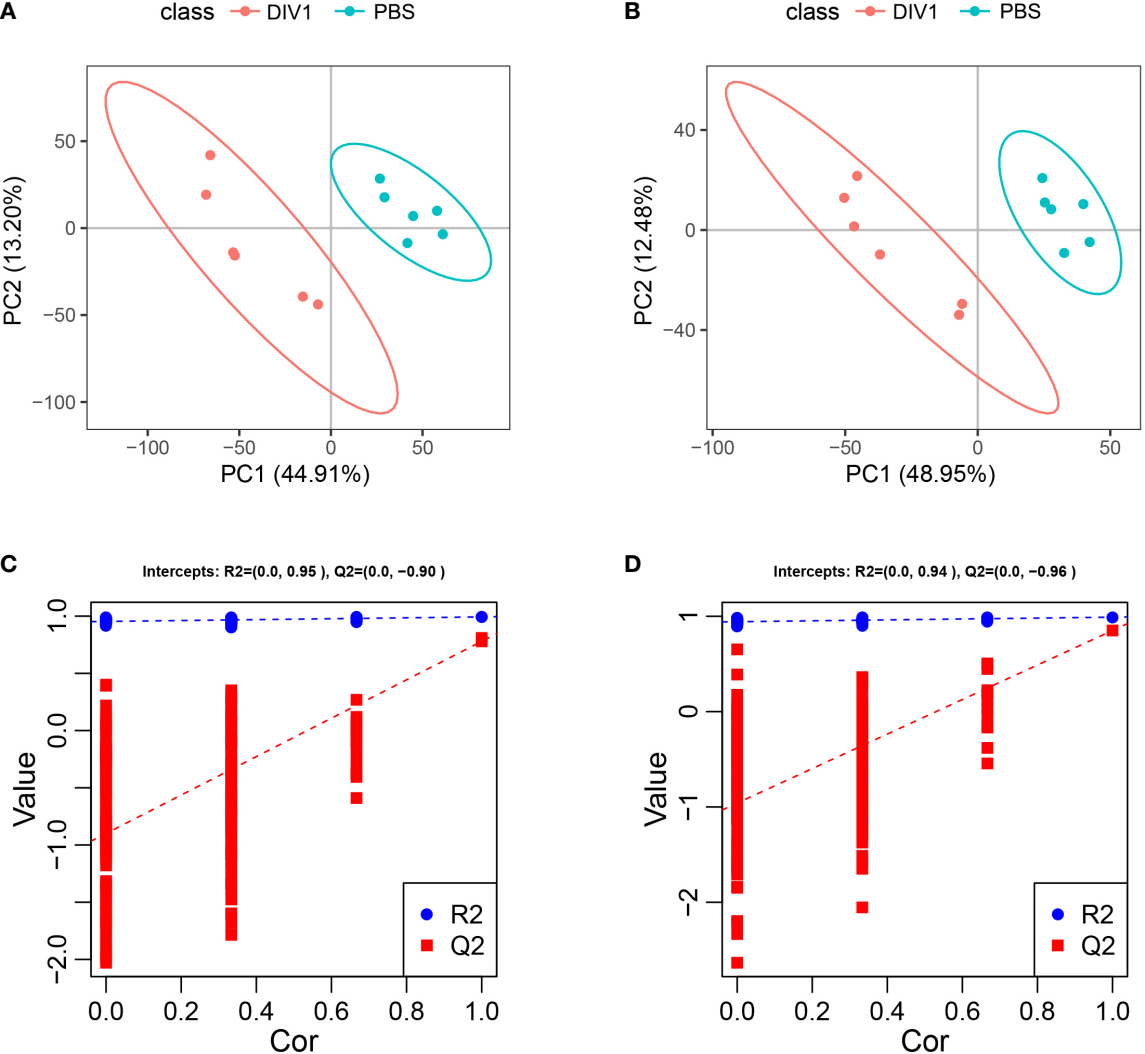
Derived PLS-DA score plots and corresponding permutation testing of PLS-DA from the LC-MS metabolite profiles in the intestine of *M. japonicus* after DIV1 infection. **(A)** PLS-DA score plot of positive ion mode. **(B)** PLS-DA score plot of negative ion mode. **(C)** Permutation testing of positive ion mode. **(D)** Permutation testing of negative ion mode.

**Figure 7:**
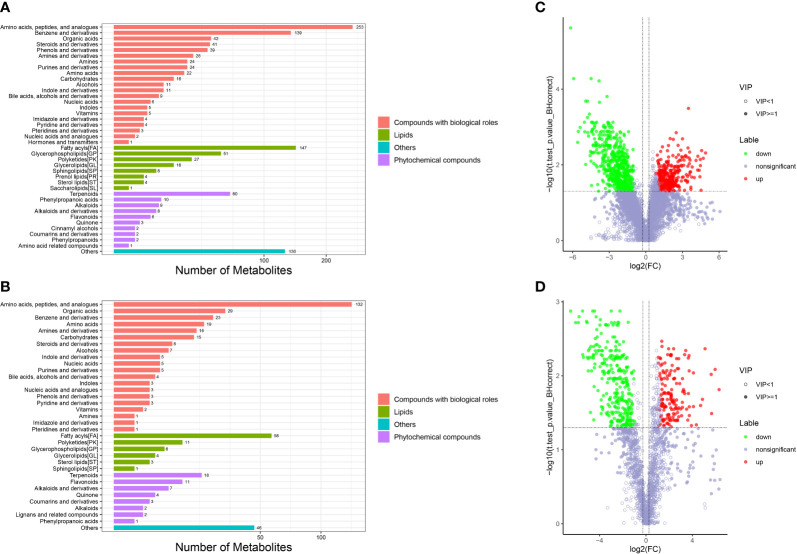
Taxonomic annotation of identified metabolites and volcano plots of DMs from the intestine of *M. japonicus*. **(A)** Taxonomic annotation of positive ion mode. **(B)** Taxonomic annotation of negative ion mode. Others means that the classification information is other categories, and the identified metabolites without classification information do not participate in the statistics. **(C)** Volcano plot of positive ion mode. **(D)** Volcano plot of negative ion mode.

Metabolomic analysis was conducted to explore the alterations in intestinal metabolic profiles after DIV1 infection. The PLS-DA score plot and permutation test showed a significant different between the two groups in both positive and negative ion mode (**Figure 6**), suggesting that DIV1 infection caused metabolic phenotype alterations in shrimp intestine. A total of 3,009 metabolites were identified in the shrimp intestine (including 2,096 metabolites were identified in the positive ion mode and 913 metabolites were identified in the negative ion mode). The classification results of the identified metabolites were shown in **Figure 7**. The largest category in the positive ion mode was “amino acids, peptides, and analogues” (253 metabolites), followed by “fatty acyls” (147 metabolites) and “benzene and derivatives” (139 metabolites) (**Figure 7A**), and the largest category in the negative ion mode was “amino acids, peptides, and analogues” (132 metabolites), followed by “fatty acyls” (58 metabolites) and “organic acids” (29 metabolites) (**Figure 7B**).

#### 3.4.2 Identification and functional annotation of the DMs

The DMs between the PBS-injected group and the DIV1-infected group were identified by the PLS-DA model with a cut-off VIP ≥ 1 and *q*-value ≤ 0.05. In the positive ion mode, a total of 868 DMs were obtained, including 312 up-regulated DMs and 556 down-regulated DMs, of which 419 DMs were identified in the database (**Figure 7C**). In the negative ion mode, a total of 454 DMs were obtained, including 158 up-regulated DMs and 296 down-regulated DMs, of which 209 DMs were identified in the database (**Figure 7D**).

**Figure 8:**
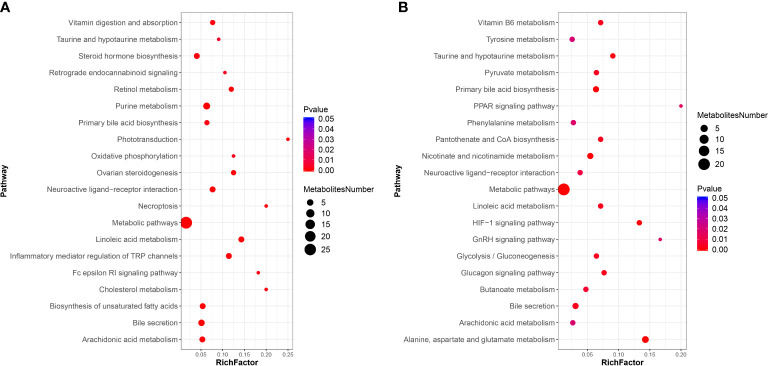
KEGG pathway enrichment analysis of DMs between the PBS-injected group and the DIV1-infected group **(A)** positive ion mode. **(B)** negative ion mode.

For the KEGG pathway enrichment analysis, the top 20 KEGG pathway enrichments influenced by DIV1 infection in the positive ion mode and negative ion mode were shown in **Figures 8A, B** respectively. Among of them, four KEGG pathways related to vitamin metabolism were significantly enriched, including “vitamin digestion and absorption”, “retinol metabolism”, “vitamin B6 metabolism” and “pantothenate and CoA biosynthesis”. Three KEGG pathways related to amino acid metabolism were significantly enriched, including “tyrosine metabolism”, “phenylalanine metabolism” and “alanine, aspartate and glutamate metabolism”. Two Warburg effect marker pathways were significantly enriched, including “pyruvate metabolism” and “glycolysis/gluconeogenesis”. Both in positive ion mode and in negative ion mode, “primary bile acid biosynthesis”, “bile secretion”, “metabolic pathways”, “linoleic acid metabolism” and “arachidonic acid metabolism” were significantly enriched in KEGG pathway enrichment analysis.

Further, in the positive ion mode, total of 41 known DMs were significantly enriched, including 25 up-regulated DMs and 16 down-regulated DMs (**Table 2**); in the negative ion mode, a total 24 DMs were significantly enriched, including 17 up-regulated DMs and 7 down-regulated DMs (**Table 3**). It was worth noting that seven types of lipid fatty acids were significantly changed after DIV1 infection, including arachidonic acid, eicosapentaenoic acid, docosapentaenoic acid, 9-oxo-10(e),12(e)-octadecadienoic acid, gamma-linolenic acid, 8(s)-hydroxy-(5z,9e,11z,14z) eicosatetraenoic acid and 16-hydroxyhexadecanoic acid. In addition, two Warburg effect marker metabolites were significantly up-regulated, including L-(+)-lactic acid and Pyruvic acid.

**Table 2:** 41 known differential metabolites were significantly enriched in KEGG pathway enrichment analysis in positive ion mode.

No.	Metabolites	log2FC	*p*-value	*q*-value	VIP	regulated
1	Succinic acid	3.493788215	0.0000	0.0000	2.0951	up
2	Deoxyguanosine monophosphate	3.319487878	0.0020	0.0020	1.5953	up
3	Retinol	2.6540001	0.0027	0.0027	1.7192	up
4	Xanthine	2.642447432	0.0001	0.0031	1.7224	up
5	Xanthosine	2.521980398	0.0008	0.0008	1.5769	up
6	(+/-)-2-hydroxyglutaric acid	2.450063094	0.0064	0.0064	1.4260	up
7	Uric acid	2.233335903	0.0028	0.0028	1.2898	up
8	Guanosine monophosphate	2.17280751	0.0037	0.0037	1.4209	up
9	Leukotriene e4	2.039805179	0.0082	0.0082	1.3434	up
10	Phenethylamine	1.922426225	0.0084	0.0084	1.3140	up
11	Icomucret	1.853875916	0.0046	0.0290	1.3534	up
12	Hypoxanthine	1.849358809	0.0036	0.0036	1.2915	up
13	All-trans-retinal	1.80467314	0.0005	0.0005	1.4709	up
14	Thymine	1.758388465	0.0038	0.0038	1.2088	up
15	Leukotriene b4	1.715102636	0.0035	0.0252	1.4015	up
16	(9cis)-retinal	1.68063942	0.0066	0.0066	1.2967	up
17	Glutarate	1.655260226	0.0025	0.0025	1.2688	up
18	Phosphoric acid	1.548979075	0.0012	0.0012	1.2447	up
19	5'-methylthioadenosine	1.523863009	0.0036	0.0036	1.4736	up
20	Arachidonic acid	1.456017576	0.0011	0.0011	1.1912	up
21	Eicosapentaenoic acid	1.344089921	0.0033	0.0033	1.1334	up
22	Androstanolone	1.310805431	0.0071	0.0071	1.0906	up
23	Docosapentaenoic acid	1.266636643	0.0074	0.0074	1.0680	up
24	Anandamide	1.260989139	0.0078	0.0390	1.0622	up
25	L-valine	1.172743517	0.0066	0.0066	1.0401	up
26	Progesterone	0.830093032	0.0018	0.0168	1.0147	up
27	Taurine	-1.415422269	0.0000	0.0013	1.3254	down
28	D-sphingosine	-1.416192117	0.0026	0.0026	1.0329	down
29	Estriol	-1.481968507	0.0037	0.0037	1.4376	down
30	9-oxo-10(e),12(e)-octadecadienoic acid	-1.717382279	0.0023	0.0023	1.3412	down
31	Gamma-linolenic acid	-1.779917739	0.0000	0.0000	1.5127	down
32	5α-dihydrotestosterone	-1.974262439	0.0006	0.0006	1.4595	down
33	Taurolithocholic acid sulfate	-2.120921629	0.0020	0.0020	1.4804	down
34	Taurocholic acid	-2.138600389	0.0009	0.0009	1.5635	down
35	Testosterone	-2.200249538	0.0008	0.0008	1.5711	down
36	Cholecalciferol	-2.321206928	0.0002	0.0002	1.6205	down
37	Phytosphingosine	-2.868805648	0.0000	0.0000	1.8610	down
38	(+/-)12(13)-dihome	-2.950090478	0.0000	0.0000	1.8567	down
39	Taurochenodeoxycholic acid	-3.604717796	0.0008	0.0008	1.9053	down
40	Kaempferol	-4.120294234	0.0000	0.0000	2.2115	down
41	3,4-dihydroxyphenylacetic acid	-4.618827395	0.0000	0.0000	2.3781	down

**Table 3:** 24 known differential metabolites were significantly enriched in KEGG pathway enrichment analysis in negative ion mode.

No.	Metabolites	log2FC	*p*-value	*q*-value	VIP	regulated
1	Cytidine	3.146492307	0.0011	0.0109	1.7973	up
2	Pantothenic acid	2.722553458	0.0069	0.0314	1.3379	up
3	Pyridoxine	2.709555326	0.0098	0.0394	1.3698	up
4	N-acetyl-l-phenylalanine	2.593234239	0.0055	0.0275	1.4078	up
5	Argininosuccinic acid	2.556306784	0.0053	0.0271	1.3755	up
6	Succinic semialdehyde	2.279560440	0.0037	0.0224	1.3252	up
7	Uric acid	2.258820397	0.0005	0.0072	1.4234	up
8	8(s)-hydroxy-(5z,9e,11z,14z)-eicosatetraenoic acid	2.183581791	0.0027	0.0191	1.3515	up
9	Phosphoric acid	2.161629747	0.0006	0.0085	1.4491	up
10	Adenine	1.969196548	0.0076	0.0333	1.1510	up
11	L-(+)-lactic acid	1.909043413	0.0023	0.0172	1.2639	up
12	Niacin	1.536649754	0.0141	0.0473	1.1171	up
13	Pyruvic acid	1.437494091	0.0040	0.0236	1.0153	up
14	Terephthalic acid	1.360645202	0.0001	0.0034	1.0953	up
15	Taurine	1.331303934	0.0080	0.0344	1.0127	up
16	Arachidonic acid	1.306845500	0.0025	0.0182	1.0367	up
17	N-acetyl-1-aspartylglutamic acid	1.085968454	0.0004	0.0067	1.0802	up
18	N-acetyl-d-mannosamine	-1.046027092	0.0011	0.0110	1.0350	down
19	16-hydroxyhexadecanoic acid	-1.569179503	0.0015	0.0129	1.3084	down
20	Cholic acid	-2.838563734	0.0000	0.0021	1.6745	down
21	(+/-)12(13)-dihome	-2.975964065	0.0000	0.0019	1.7010	down
22	Luteolin	-3.930160375	0.0000	0.0017	1.9612	down
23	Taurochenodeoxycholic acid	-4.316168826	0.0001	0.0038	2.1122	down
24	N-acetylmuramic acid	-4.952322025	0.0001	0.0046	1.9742	down

### 3.5 Association between the altered metabolites and microbial

**Figure 9:**
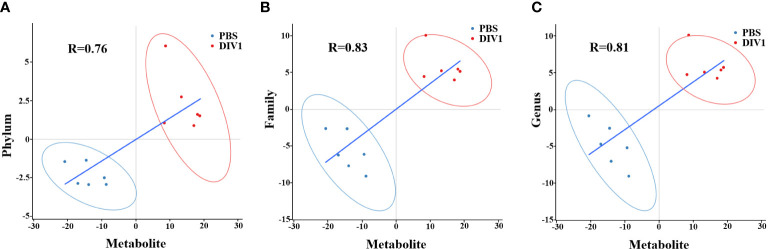
Scatter plots of correlation between DMs and hosts intestinal microbial. **(A)** Phylum classification level. **(B)** Family classification level. **(C)** Genus classification level.

**Figure 10:**
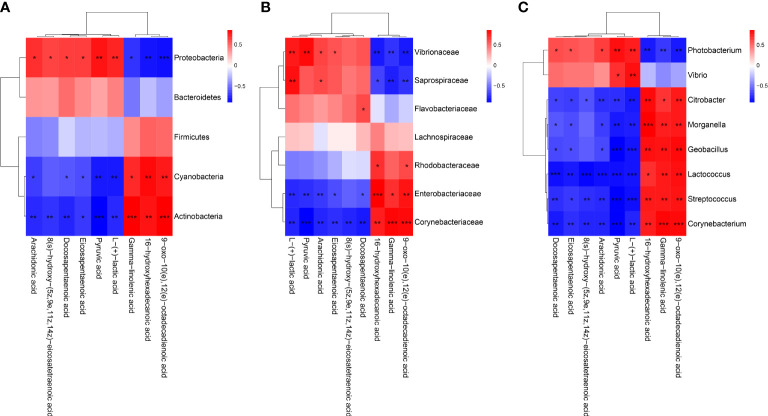
Heap maps of correlation between the nine marker DMs and the hosts intestinal microbial. **(A)** Phylum classification level. **(B)** Family classification level. **(C)** Genus classification level. Red indicateds a positive coreelation, and blue represents a negative correlation. * Indicates a significant difference (*p < 0.05; **p < 0.01; ***p < 0.001).

To reveal the relationships between intestinal microbial and DMs, scatterplots at the phylum, family, and genus levels were generated by canonical correlation analysis (**Figure 9**). The results showed that there was a strong correlation (R value > 0.75) between the host intestinal microbial and DMs at all taxonomic levels, and the samples from different groups had a large degree of dispersion. To further analyze the relationship between the nine marker DMs (including seven types of fatty acids and two Warburg effect marker metabolites) and host intestinal microbial, heat maps at the phylum, family, and genus levels were generated by spearman correlation analysis (**Figure 10**). The results showed that, at the phylum classification level, Proteobacteria was positively associated with arachidonic acid, 8(s)-hydroxy-(5z,9e,11z,14z) eicosatetraenoic acid, docosapentaenoic acid, eicosapentaenoic acid, pyruvic acid and L-(+)-lactic acid, and negatively associated with gamma-linolenic acid, 16-hydroxyhexadecanoic acid and 9-oxo-10(e),12(e)-octadecadienoic acid. The association of Cyanobacteria and Actinobacteria with marker metabolites was completely opposite to that of Proteobacteria, while neither Bacteriodetes nor Firmicutes were significantly associated with the marker metabolites (*p* > 0.05). It was worth noting that, *Photobacterium* and *Vibrio* in the Vibrionaceae that dominated the DIV1-infected group, were positively associated with Warburg effect marker metabolites L-(+)-lactic acid and pyruvic acid. In contrast, *Corynebacterium* in the Corynebacteriaceae that dominated the PBS-injected group, was negatively correlated with L-(+)-lactic acid and pyruvic acid. This phenomenon also appeared in the correlation with fatty acid metabolites.

## 4 Discussion

Growing evidences suggested that viral infection or environmental stress can lead to changes in the structure and function of the host’s intestinal microbiota, and further affect the normal metabolism of the host and caused various adverse reactions (28–31). To date, there are no reports on the effects of DIV1 infection on the intestinal microbiota and metabolites of *M. japonicus*. The present study provides insights into the interaction of *M. japonicus* and DIV1 through the histological analysis, enzyme activity analysis and the integrated analysis of intestinal microbiome and metabolomics.

The results of histological analysis showed that severe intestinal mucosal damage was observed in the intestine of DIV1-infected shrimp, some intestinal epithelial cells were detached from the basement membrane and the intestinal villus height was significantly reduced. Similarly, Xue et al. found that WSSV-infected crayfish *Procambarus clarkii* exhibited worse intestinal histomorphology, with thinner intestinal walls and shorter intestinal villi compared to healthy crayfish (32). Liang et al. found that the intestinal epithelial cells of *L. vannamei* infected with *Vibrio parahaemolyticus* were completely detached from the basement membrane (33). Except for the intestinal mucosal damage, the disturbance of intestinal immunity and digestive function was also observed in the DIV1-infected shrimp. Enzyme activity analysis showed that all digestive enzymes and immune enzymes were significantly altered (*p* < 0.001), indicating that DIV1 infection severely affected the normal immune and digestive functions of the *M. japonicus* intestine. Studies have found that shrimp hemocytes can produce a large amount of free radicals and reactive oxygen species (ROS) during phagocytosis, thereby effectively killing the invading pathogens (34, 35). However, the mass accumulation of ROS in animals will cause serious cell damage, resulting in various diseases (36). SOD and CAT are two kinds of antioxidant enzymes, which can effectively remove free radicals and ROS, thereby protecting cells from oxidative stress (37–39). Under the action of SOD, ROS react with hydrogen ions to generate less toxic hydrogen peroxide, and then react with hydrogen ions under the action of CAT to finally produce harmless water (40). Therefore, only when the activities of SOD and CAT are coordinated, the free radicals and ROS in the organism can be maintained at a low level. However, the trends in the activity of SOD and CAT were not always consistent. In *L. vannamei*, intramuscular injection of 0.1 μg (g shrimp)^−1^ fluorescent red polyethylene microspheres (41) and experimental infection with *Enterocytozoon hepatopenaei* (42) both resulted in a significantly increase in SOD activity and a significant decrease in CAT activity. In *Strongylocentrotus intermedius*, the activity of SOD increased significantly and the activity of CAT decreased significantly with increasing temperature (43). In our study, the activities of SOD was significantly increased in the intestine of the *M. japonicus* under DIV1 infection, while the activities of CAT was significantly decreased. These results are supported by the previous findings, and suggested that DIV1 infection may cause a large amount of intestinal reactive oxygen species to be produced and inhibit the antioxidant capacity of the intestine to some extent. The specific mechanism needs to be further studied. LYZ has been shown to play an important role in shrimp resistance to viral and bacterial infections (44–46). The activity of LYZ can directly reflect the strength of shrimp immune function. After infection with DIV1, the activity of LYZ in the intestine of *M. japonicus* was significantly decreased, which indicated that the intestinal immune function was suppressed to some extent. α-AMS, LPS and TPS are three important digestive enzymes that also serve as markers to help diagnose diseases. Several studies had reported that the significantly increased α-AMS and LPS activities can be used as the basis for the diagnosis of mammalian enteritis and pancreatitis (47–51), while trypsin deficiency is closely related to enteritis (52–54). In this study, the intestinal activities of α-AMS and LPS were significantly increased after infection with DIV1, while the activities of TPS was significantly decreased. This suggested that DIV1 infection may cause damage to the shrimp intestine, causing enteritis and digestive dysfunction. In addition, this may also be one of the reasons for the decreased feeding rate of DIV1-infected shrimp and caused the symptoms of empty stomach and intestine (14, 25, 26, 55, 56).

In intestinal microbiome analysis, after DIV1 infection at 24 h, the intestine microbiome was significantly changed in the composition and diversity at the phylum, family and genus levels. In this study, the intestinal microbiota in *M. japonicus* were dominated by five phyla of Proteobacteria, Actinobacteria, Bacteroidetes, Firmicutes and Cyanobacteria in both the PBS-injected group and DIV1-infected group, which is consistent with the previous microbiome studies in *L. vannamei*. At the family level, the relative abundance of Vibrionaceae significantly increased in the intestine of DIV1-infected *M. japonicus* compared with healthy *M. japonicus*, which mainly reflected in the significant increase in the relative abundance of *Photobacterium* and *Vibrio* at the genus level. In addition, both the LEfSe cladogram and LDA score of LEfSe-PICRUSt showed that *Photobacterium* and *Vibrio* dominated in the DIV1-infected group. Several previous studies had shown that WSSV infection can cause an increase in the relative abundance of opportunistic pathogens in the intestine of shrimp, such as *Aeromonas*, *Arcobacter*, *Vibrio* and *Trichococcus* (19, 21, 32). *Photobacterium* had been shown to cause various diseases in fish and shrimp (57–61) causing huge losses in aquaculture. *Vibrio*, which is generally considered to be an opportunistic pathogen of shrimp and widely exists in the intestine of shrimp, can cause shrimp disease when the environment changes drastically (62–65). These results suggested that DIV1 infection can cause an increased abundance of opportunistic pathogens in the intestine, which may probably due to DIV1 infection disrupting the mechanical barrier of the intestine and impairing the ability of intestine to select microorganisms. The results of PICRUSt functional prediction showed that, in the KEGG level 2, the relative abundance of “carbohydrate metabolism”, “metabolism of cofactors and vitamins” and “amino acid metabolism” were the top 3 in the two groups. It meant that intestinal microbiota played an important role in regulating host vitamin metabolism, carbohydrate metabolism and amino acid metabolism. Notably, the relative abundance of “infectious diseases: bacterial” was significantly increased under DIV1 infection, which further suggested that DIV1 infection could cause secondary bacterial infection. In-depth analysis of the top 3 KEGG level 2 terms showed that the abundance value of amino acid metabolism, vitamin metabolism, glycolysis/gluconeogenesis and pyruvate metabolism were significantly increased after DIV1 infection. It meant that DIV1 can promote the Warburg effect by affecting the function of the host intestinal microbiota.

A recent study found that the “linoleic acid metabolism” and “arachidonic acid metabolism” were also the most disturbed pathways by HCoV-299E infection (66). Interestingly, exogenous supplement of linoleic acid or arachidonic acid in HCoV-229E-infected cells significantly suppressed HCoV-229E virus replication (66). Another study showed that linoleic acid could directly bound to WSSV to inhibited the viral replication and indirectly participate in the immune response against WSSV by activating the ERK-NF-κB signaling pathway to promote the expression of antimicrobial peptides and IFN-like gene Vago5 (67). Consistently, the intestinal metabolomics analysis in this study showed that lipid metabolism in the intestine of *M. japonicus* was remodeled after DIV1 infection, and a total of seven types of lipid fatty acids were significantly changed, including arachidonic acid, eicosapentaenoic acid, docosapentaenoic acid, 9-oxo-10(e), 12(e) -octadecadienoic acid, gamma-linolenic acid, 8(s)-hydroxy-(5z,9e,11z,14z) eicosatetraenoic acid and 16-hydroxyhexadecanoic acid. In addition, two Warburg effect marker metabolites were significantly up-regulated, including L-(+)-lactic acid and pyruvic acid. KEGG enrichment analysis indicated that the “Linoleic acid metabolism” and “arachidonic acid metabolism” pathways were the most affected by DIV1 infection. These results suggested that the lipid metabolic reprogramming of *M. japonicus* was significantly associated with DIV1 infection and replication. After infected with DIV1, two marker metabolites of the Warburg effect were significantly up-regulated in the shrimp intestine, including L-(+)-lactic acid and pyruvic acid, and significantly enriched in the “pyruvate metabolism” and “glycolysis/gluconeogenesis” pathways. In addition, some amino acid metabolism-related pathways were also significantly enriched under DIV1 infection, including “tyrosine metabolism”, “phenylalanine metabolism” and “alanine, aspartate and glutamate metabolism”. These phenomena also appeared in previous studies (11, 25, 26), which may be attributed to the material and energy requirements of DIV1 replication and suggested that DIV1 may contain AMGs for regulating host amino acid metabolism, carbohydrate metabolism and energy metabolism. It was worth noting that some pathways related to vitamin metabolism were significantly enriched, such as “retinol metabolism”, “vitamin B6 metabolism” and “pantothenate and CoA biosynthesis”. Retinol, also known as vitamin A (VA), plays an important and positive role in intestinal mucosal immunity and repair (68). The B vitamins can regulate the energy metabolism, nutrient accumulation and immune defense function of aquatic animals (69). Our previous study showed that DIV1 infection can regulate the vitamin metabolism of shrimp by affecting the expression of intestinal miRNA and mRNA (11, 26). In this study, from the metabolome level, it was further demonstrated that DIV1 infection can regulate vitamin metabolism in the intestine, thereby affecting the normal physiological function of *M. japonicus*.

Intestinal microbiota variation caused by viral infection or environmental stress can affect the metabolism and immunity of their host, and increase disease susceptibility (70, 71). To further explore the relationship between intestine microbiota variation and metabolites, the correlation analysis of dominant intestinal microbial and marker DMs was carried out. In this study, the increased levels of *Photobacterium* and *Vibrio* were positively correlated with changes in Warburg effect marker metabolites L-(+)-lactic acid and pyruvic acid. This indicated that *Photobacterium* and *Vibrio* may synergize with DIV1 to jointly promoted the Warburg effect, providing material and energy for the successful replication of DIV1. In addition, the abundances of *Photobacterium* and *Vibrio* were positively associated with arachidonic acid, eicosapentaenoic acid, docosapentaenoic acid and 8(s)-hydroxy-(5z,9e,11z,14z) eicosatetraenoic acid and negatively associated with 9-oxo-10(e),12(e)-octadecadienoic acid, gamma-linolenic acid and 16-hydroxyhexadecanoic acid. However, *Corynebacterium*, which dominated the PBS-injected group, was just the opposite of the above. It suggested that *Photobacterium* and *Vibrio* may be involved in the metabolic reprogramming of the shrimp intestine after DIV1 infection.

## 5 Conclusions

**Figure 11:**
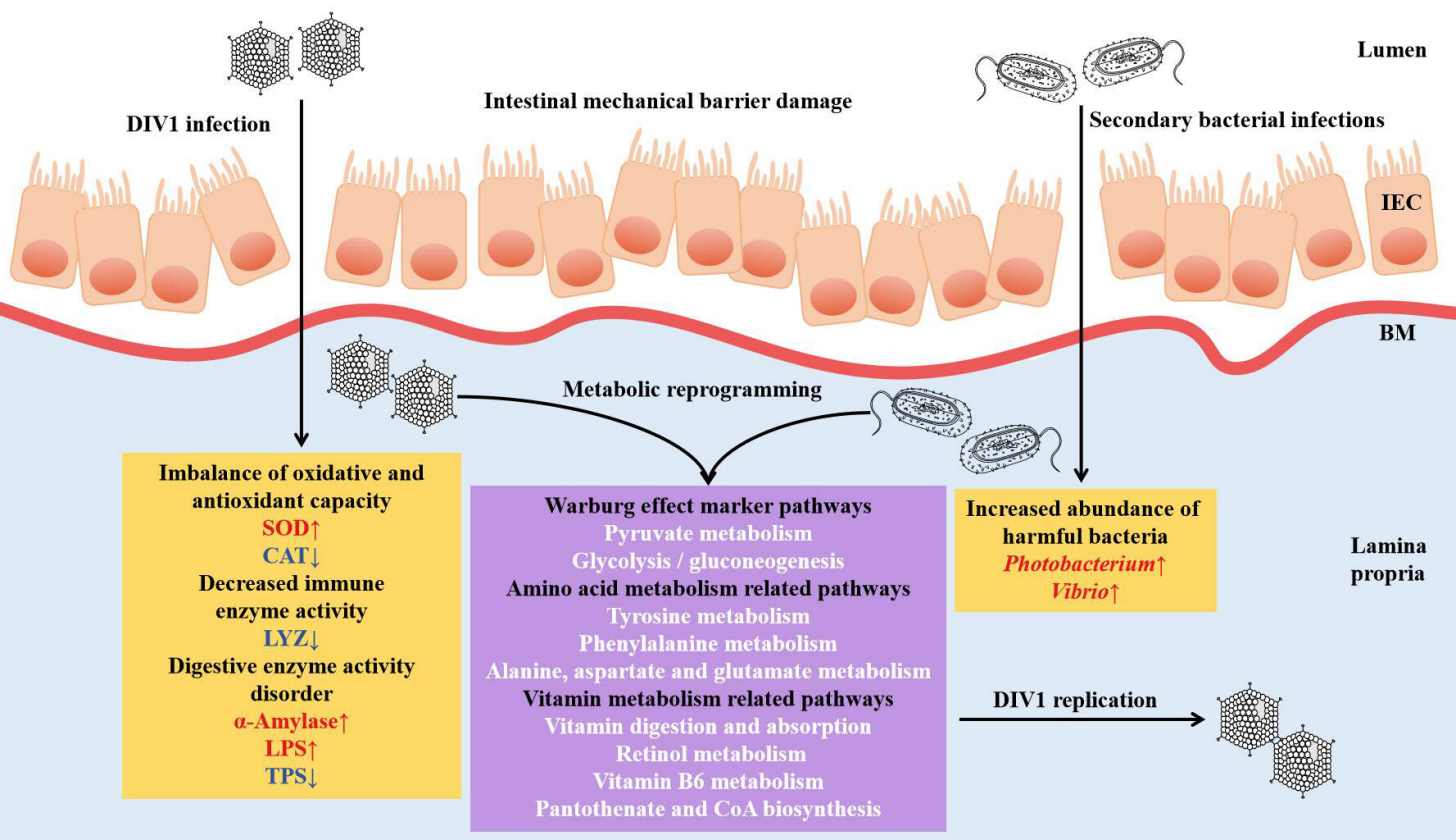
Proposed schematic diagram of secondary bacterial infection and metabolic reprogramming of *M. japonicus* induced by DIV1 infection. IEC, intestinal epithelial cells; BM, basement membrane; SOD, superoxide dismutase; CAT, catalase; LYZ, lysozyme; LPS, lipase; TPS, trypsin.

In conclusion, through the histological analysis, enzyme activity analysis and the integrated analysis of intestinal microbiome and metabolomics, the present study revealed that DIV1 infection can lead to the damage of the intestinal mechanical barrier of shrimp, the imbalance of oxidative and antioxidant capacity, the decrease of immune enzyme activities, and the disturbance of digestive enzyme activities. Futhermore, harmful bacteria may cooperate with DIV1 to promote the Warburg effect and induce metabolic reprogramming, thereby creating favorable conditions for the replication of DIV1. The speculation needs to be further verified. This study is the first to report the changes of intestinal microbiota and metabolites of *M. japonicus* under DIV1 infection, demonstrating that DIV1 can induce secondary bacterial infection and metabolic reprogramming (**Figure 11**).

## Data availability statement

The data presented in the study are deposited in the Sequence Read Archive repository, accession number PRJNA720257.

## Author contributions

ZH, SZ, and CS contributed to conception and design of the study. ZH, YZ, ML, LD, and YW collected the samples and performed the experiments. ZH analyzed the sequencing data, organized all the results, wrote the original draft, and revised the manuscript. YZ helped to perform statistical analysis, conceptual analysis and manuscript revision. SZ performed the writing review and editing. CS contributed to project administration and funding acquisition. All authors contributed to the article and approved the submitted version.

## Funding

This work was supported by the key research and development projects in Guangdong Province (Grant No. 2020B0202010009), the Science and Technology Program of Guangdong Province (Grant No. 2021B0202020003), and the project of the innovation team for the innovation and utilization of Economic Animal Germplasm in the South China Sea (Grant No. 2021KCXTD026).

## Acknowledgments

Our acknowledgments go to all funders of this work.

## Conflict of interest

The authors declare that the research was conducted in the absence of any commercial or financial relationships that could be construed as a potential conflict of interest.

## Publisher’s note

All claims expressed in this article are solely those of the authors and do not necessarily represent those of their affiliated organizations, or those of the publisher, the editors and the reviewers. Any product that may be evaluated in this article, or claim that may be made by its manufacturer, is not guaranteed or endorsed by the publisher.
